# Night-time neuronal activation of Cluster N in a day- and night-migrating songbird

**DOI:** 10.1111/j.1460-9568.2010.07311.x

**Published:** 2010-08

**Authors:** Manuela Zapka, Dominik Heyers, Miriam Liedvogel, Erich D Jarvis, Henrik Mouritsen

**Affiliations:** 1 AG “Neurosensorik”, Institut für Biologie und Umweltwissenschaften, University of OldenburgD-26111 Oldenburg, Germany; 2 Department of Ecology, Animal Ecology, Lund UniversitySölvegatan, Lund, Sweden; 3 Department of Neurobiology, Howard Hughes Medical Institute, Duke University Medical CenterDurham, NC 27710, USA

**Keywords:** bird migration, magnetic sense, magnetoperception, meadow pipit, navigation

## Abstract

Magnetic compass orientation in a night-migratory songbird requires that Cluster N, a cluster of forebrain regions, is functional. Cluster N, which receives input from the eyes via the thalamofugal pathway, shows high neuronal activity in night-migrants performing magnetic compass-guided behaviour at night, whereas no activation is observed during the day, and covering up the birds’ eyes strongly reduces neuronal activation. These findings suggest that Cluster N processes light-dependent magnetic compass information in night-migrating songbirds. The aim of this study was to test if Cluster N is active during daytime migration. We used behavioural molecular mapping based on ZENK activation to investigate if Cluster N is active in the meadow pipit (*Anthus pratensis*), a day- and night-migratory species. We found that Cluster N of meadow pipits shows high neuronal activity under dim-light at night, but not under full room-light conditions during the day. These data suggest that, in day- and night-migratory meadow pipits, the light-dependent magnetic compass, which requires an active Cluster N, may only be used during night-time, whereas another magnetosensory mechanism and/or other reference system(s), like the sun or polarized light, may be used as primary orientation cues during the day.

## Introduction

Twice each year, millions of migratory songbirds travel thousands of kilometres between their breeding grounds and overwintering sites and back, thereby using a geomagnetic compass and/or celestial cues to find their way (for a review see Wiltschko & Wiltschko, 1996). Experiments with free-flying songbirds have suggested that the magnetic compass is the primary reference system during night-time flights (Cochran *et al.*, 2004). But how do the birds sense the magnetic compass direction?

Currently, two magnetic sensor systems are supported by experimental evidence: (i) iron-mineral-based structures in the upper beak of the birds (Kirschvink *et al.*, 2001; Fleissner *et al.*, 2003, 2007; Falkenberg *et al.*, 2010), putatively acting as a part of a magnetic map/signpost sense (Mora *et al.*, 2004; Heyers *et al.*, 2010); and (ii) light-dependent radical-pair reactions in the birds’ eyes (Ritz *et al.*, 2000, 2004; Maeda *et al.*, 2008) probably providing directional compass information. The light-dependent magnetic sensing hypothesis is supported by the finding of putative receptor molecules, the cryptochromes, in the retina of night-migratory birds (Möller *et al.*, 2004; Mouritsen *et al.*, 2004b). The cryptochrome 1a found in garden warblers (*Sylvia borin*) has all the thus far testable biophysical characteristics (Liedvogel *et al.*, 2007b) required by theory (Ritz *et al.*, 2000; for a review see Liedvogel & Mouritsen, 2010).

Correct magnetic compass orientation requires that the sensory input derived from the ambient magnetic field is integrated and processed in the brain. A forebrain region, Cluster N, was shown to be highly active in two night-migratory species, European robins (*Erithacus rubecula*) and garden warblers, at night but not in two non-migrating songbird species, zebra finches (*Taeniopygia guttata*) and canaries (*Serinus canaria*), tested under the same conditions (Mouritsen *et al.*, 2005). This neuronal activation of Cluster N is movement independent (Feenders *et al.*, 2008) and disappears when the eyes of the birds are covered (Mouritsen *et al.*, 2005; Liedvogel *et al.*, 2007a). Furthermore, a link between the eyes and Cluster N via the thalamofugal visual pathway has been documented (Heyers *et al.*, 2007). Together, these data strongly suggested that Cluster N is involved in light-mediated magnetoreception, and this was recently confirmed with lesion studies: European robins with chemically inactivated Cluster N were no longer able to perform correct magnetic compass orientation, whereas the birds’ star and sun compass orientation remained unaffected (Zapka *et al.*, 2009). Cluster N therefore seems crucial for the processing of magnetic compass information in night-migrants, but is Cluster N also used during the day?

Several studies on day-migratory birds, most of which also migrate during the night (Alerstam, 1990), revealed that they can also use a magnetic compass (e.g. Munro & Wiltschko, 1993; Gudmundsson & Sandberg, 2000). The main objective of this study was thus to investigate whether and when Cluster N is activated in birds that migrate both during day- and night-time, like the meadow pipit (*Anthus pratensis*).

We analysed neuronal activity using sensory-driven expression of the immediate early gene ZENK in Cluster N of meadow pipits that experienced the local geomagnetic field either during the day or the night.

## Material and methods

### Animals

All animal procedures were performed in accordance with local and national guidelines for the use of animals in research and were approved by the appropriate authorities (Niedersächsisches Landesamt für Verbraucherschutz und Lebensmittelsicherheit). Twelve day-migratory meadow pipits were wild-caught at the North Sea coast, Germany, 35 km north of Oldenburg, in autumn 2006, and were housed indoors under local photoperiod conditions. Magnetic field exposures were conducted during the non-migratory season between 15 December 2006 and 17 January 2007. Mouritsen *et al.* (2005), Liedvogel *et al.* (2007a) and Feenders *et al.* (2008) have shown that time of the year had no effect on ZENK expression in Cluster N. This parallels other sensory systems such as the songbird auditory system, where the auditory centres of males of a seasonal species are activated by conspecific male song at all times of the year, despite the fact that conspecific male songs only elicit strong behaviour during spring time (Jarvis *et al.*, 1995).

### Behavioural test procedures

The behavioural set-up and procedures used in this study corresponded to those described previously (Mouritsen *et al.*, 2005). The experiments took place in a wooden house, where the birds had undisturbed access to the Earth’s magnetic field. During the experiment, the bird was placed in a cylindrical, transparent Plexiglas cage (height 40 cm, diameter 40 cm) fitted with a circular perch (8.5 cm above the ground) (Mouritsen *et al.*, 2004a).

To document the bird’s movements, a small stripe of reflective tape was glued to each bird’s head. In order to allow the birds to get used to the experimental set-up, the birds were individually placed into the Plexiglas cage 3 h before the behavioural experiment started. The birds were either tested during the day under full room light (250 mW/m^2^) or during the beginning of the night under dim light provided by eight small light bulbs on the floor (4 mW/m^2^). During this time, each bird was carefully observed via two infrared-sensitive cameras (top- and side-view, 840 nm), connected to a surveillance monitor, and recorded to video. The birds were killed after they had been sitting relatively still but awake for at least 45 min in the cage while a minimum of other behaviours occurred (i.e. 0–20 flights; 0–10 jumps up and down the perch), thereby minimizing motor activity-dependent gene induction in the brain (Feenders *et al.*, 2008). Birds were decapitated, the brains extracted, the two hemispheres separated, embedded in Tissue-Tek O.C.T. (Sakura Finetek, Zoeterwoude, the Netherlands) and quick frozen in a dry ice/ethanol bath to −80°C all within 5–12 min to avoid detection of stress-induced ZENK mRNA expression.

### Gene expression analyses

To link specific sensory inputs to brain activity patterns, we used behavioural molecular mapping. We measured the expression of ZENK [acronym for zif28, Egr1, NGF-1A, Krox-24], an immediate early gene (IEG) in the brain. ZENK expression is driven by neuronal activity and ZENK mRNA can be detected about 15 min after onset of neuronal firing with peak expressions after 30–45 min (Jarvis & Nottebohm, 1997). ZENK is expressed in most parts of the bird brain, except in primary thalamic recipient neurons of the forebrain, the globus pallidus and parts of the thalamus (Jarvis, 2004). ZENK mRNA staining labels brain regions that were active during the last 15–60 min of a specific behaviour or sensory stimulation (Mello *et al.*, 1992; Mello & Clayton, 1995; Jarvis & Nottebohm, 1997). Thus, in our experiment, the expression pattern of the accumulated IEG mRNA mirrors the neuronal activity pattern in the brain during the last 45–60 min before the tissue was fixed. To detect the ZENK mRNA expression pattern, we used radioactive *in situ* hybridization following a previously described protocol (Wada *et al.*, 2006). We cut the left hemisphere in 12-μm sagittal sections, collected as ten parallel series, fixed the brain slices with 3% paraformaldehyde and hybridized them with S35-UTP riboprobes made from zebra finch cDNA. The hybridized sections were exposed to X-ray film (Biomax, Kodak) for 1–3 days and then dipped into autoradiographic photoemulsion (NTB2, Kodak) for 4–6 weeks at 4°C, developed (Kodak developer D19; Kodak fixer, Kodak), Nissl stained with cresyl violet acetate (Sigma, Deisenhofen, Germany) and coverslipped with permount glue (Fisher Scientific, Loughborough, UK).

### Immunohistochemistry

Ionotropic glutamate receptor type 1 (GluR1) was used as an anatomical marker to verify the borders of avian brain subdivisions (Reiner *et al.*, 2004; Mouritsen *et al.*, 2005) in the meadow pipit; here we analyse the protein (Wada *et al.*, 2004) instead of the mRNA. Two birds were killed by intramuscular injection of an overdose of Narcoren (Merial, Hallbergmoos, Germany). The tissue was fixed by transcardial perfusion with 0.12 m phosphate-buffered saline (PBS) containing 0.1% heparin sodium salt (Sigma) followed by 4% paraformaldehyde (PFA) dissolved in PBS. Whole brains were dissected from the skull and postfixed in 4% PFA dissolved in PBS for 3 h. The tissue was cryoprotected in 30% sucrose (dissolved in PBS) for 24 h and cut into 40-μm sagittal sections. The sections were stored in PBS containing 0.01% Na-azide at 4°C before staining. The brain sections were stained in free-floating reactions according to the immuno-ABC technique (ABC Elite Kit Rabbit IgG, Catalog #PK-6101, Vector Laboratories, Burlingame, CA, USA). Each incubation step was followed by three PBS rinses lasting 5 min each. Endogenous peroxidases were inactivated by incubation with 0.3% hydrogen peroxide dissolved in distilled water for 60 min, and unspecific binding sites were blocked by incubating the slices in 10% fetal calf serum (Kraeber, Ellerbek, Germany) for 60 min. Slices were then incubated with a rabbit polyclonal GluR1 (Temecula, CA, USA; product no. AB1504, Lot 24010521; working dilution: 1:1000) antibody overnight at 4°C with gentle agitation. After washing, sections were incubated for 60 min with an appropriate biotinylated secondary antibody and avidin-coupled peroxidase-complex (Vector ABC Elite kit; Vector Laboratories, Burlingame, CA, USA). Peroxidase activity was detected using a 3′3-diaminobenzidine (DAB; Sigma) reaction, modified by using β-d-glucose/glucose-oxidase (Sigma; Shu *et al.*, 1988). After sufficient reaction product was formed, the reaction was stopped in PBS. Sections were mounted on gelatinized glass slides, dehydrated in an ascending series of ethanol (70, 96, 100%) followed by xylene and embedded in Entellan (Merck, Darmstadt, Germany).

### Analysis, digital processing and photomicrograph production

Images of the relevant brain sections from each individual were taken with a digital camera (Leica DFC320, Solms, Germany) connected to a stereomicroscope (Leica M, Leica IM 50, Solms, Germany). X-ray films of brain sections and immunohistochemically stained brain sections were documented as bright-field images, adjusted to have the same contrast and brightness, and served as figures used in this article; no additional filtering or manipulation of the images was performed. Dark staining in the form of silver grains reflect high levels of ZENK mRNA expression. To quantify the darkness (and thus ZENK expression level) of a region, a person naïve to the experimental conditions used the anatomical boundaries visible in the *in situ* hybridized slides and the GluR1-stained sections to encircle the brain regions of interest with a pen display (Wacom Cintiq 21UX, Krefeld, Germany) in Photoshop 7.0/Illustrator 10.0 software (Adobe Systems, San Jose, CA, USA). The mean pixel density was measured using the 256-level greyscale of the ‘Histogram’ function in Adobe Photoshop 7.0 (Wada *et al.*, 2004; Mouritsen *et al.*, 2005). In addition to the direct quantification of the mean pixel density in Cluster N, we also controlled for any potential differences in the background staining intensity by using the same procedure as in prior studies (Mouritsen *et al.*, 2005; Liedvogel *et al.*, 2007a): we quantified the mean pixel density of the posterior dorsal parts of the hyperpallium (H), interstitial region of the hyperpallium (IH) and dorsal mesopallium (MD) (i.e. Cluster N) relative to the mean pixel density of the anterior ventral part of the H, IH and MD (a control region irrelevant to magnetic sensing) by subtracting the latter from the former. We used the sigmastat 3.0 software package (Aspire Software International, Ashburn, VA, USA) to test for significant differences between day- and night-groups. Neuroanatomical structures were named according to the revised nomenclature for the avian telencephalon (Reiner *et al.*, 2004; Jarvis *et al.*, 2005) with modifications (Feenders *et al.*, 2008).

## Results

**FIG. 1:**
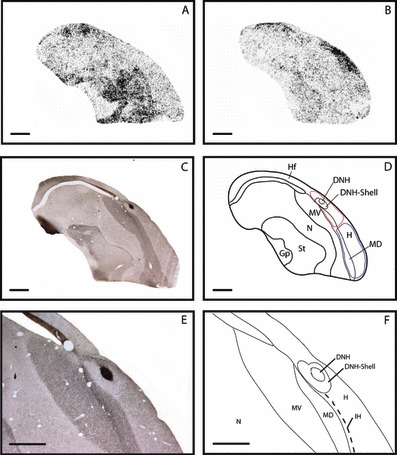
Autoradiographs showing ZENK brain activation during (A) daytime and (B) night-time in day- and night-migrating meadow pipits. During night-time (B) high neuronal activity, indicated by ZENK expression, occurred in Cluster N, whereas during the day (A) Cluster N does not show increased neuronal activity. Instead, during the day, brain regions, comprising parts of MV, N and St known to be active during movement and to process day vision (lateral to this section) are highly active. ZENK expression indicating neuronal activity: black dots. (C) GluR1 staining for anatomical characterization. (D) Anatomical profile of a parasagittal section; red and blue lines indicate the regions quantified for Fig. 2. (E) Higher magnification of GluR1 staining in and around Cluster N. (F) Detailed anatomical profile of Cluster N (parasagittal brain sections are shown). Dorsal is up; anterior is right; scale bar = 1.5 mm. Abbreviations: Gp, globus pallidus; St, striatum; N, nidopallium; MD, dorsal mesopallium; MV, ventral mesopallium; H, hyperpallium; Hf, hippocampal formation; IH, interstitial layer of the hyperpallium; DNH, dorsal nucleus of the hyperpallium.

We analysed ZENK expression patterns in the forebrain of meadow pipits. The birds were tested under two different conditions: during the day with full room light (*n* = 6) and during the night under dim light (*n* = 4). Relative to the day (Fig. 1A), meadow pipits tested during the night showed high ZENK expression in a distinct cluster of brain regions located in the hyperpallium and mesopallium (Fig. 1B) that is comparable with the neuroanatomical location of Cluster N in night-migratory European robins and garden warblers (Mouritsen *et al.*, 2005). The remainder of the forebrain showed low to no ZENK expression. During the day, motor areas of the anterior forebrain (anterior MV, N, and St) had high ZENK expression levels in animals that moved a lot (Fig. 1A), as expected given that these are motor-associated brain areas (Feenders *et al.*, 2008).

To characterize the anatomical location of the active brain regions more accurately and to distinguish the boundaries between brain subdivisions, a series of brain sections from two different birds was stained with cresyl violet (Nissl stain; data not shown) and GluR1 (Fig. 1C). The Nissl- and GluR1-staining results were identical to the staining results in and around Cluster N seen in all other songbird species we and others have investigated so far using mRNA *in situ* and various brands of ionotropic GluR1 antibodies. Nissl- and GluR1-staining showed that the area active during night-time in meadow pipits corresponds to Cluster N in night-migrants: it includes parts of the H, IH, the MD, the dorsal nucleus of the hyperpallium (DNH), and a shell-like structure around the DNH (Fig. 1D–F). It extends approximately 1.4 mm rostrocaudal, 1.6 mm dorsoventral and 1.6 mm mediolateral. This corresponds to the neuroanatomical boundaries of Cluster N in night-migratory European robins and garden warblers (Mouritsen *et al.*, 2005; Liedvogel *et al.*, 2007a), showing that the part of the forebrain that is active at night in meadow pipits is Cluster N.

**FIG. 2:**
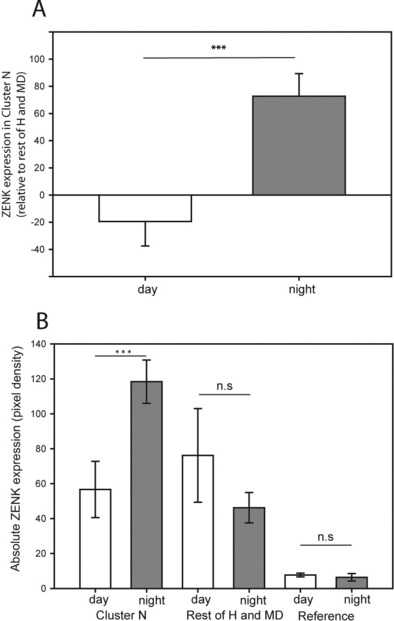
Quantification of ZENK expression expressed as pixel density on a 256 grey scale (white = 0, black = 255). (A) Relative ZENK expression in Cluster N (i.e. the posterior dorsal parts of the H, IH, MD) minus the anterior ventral part of the H, IH and MD) was significantly higher in meadow pipits during night-time and dim-light conditions compared with meadow pipits exposed to bright light during the day. (B) Differences in absolute ZENK expression between birds tested during day and night were highly significant in Cluster N but not in the rest of H+MD or in the globus pallidus. ***P < 0.001; n.s = not significant. Abbreviations: MD, dorsal mesopallium; H, hyperpallium; IH, interstitial layer of the hyperpallium.

Cluster N showed high ZENK expression in birds tested during the night [mean pixel density 118 ± 16 (SD)]. During the day, ZENK expression in Cluster N (mean pixel density 57 ± 12) was significantly lower (*t*-test: d.f. = 8, *t* = 6.45, *P* < 0.001; Mann–Whitney *U*-test: *n*_1_ = 6, *n*_2_ = 4, *U* = 24, *P* = 0.01) (Fig. 2B). ZENK activation in Cluster N was significantly higher in the night-time group than in the daytime group both when the absolute pixel densities were considered and when the ZENK expression in Cluster N was related to the ZENK expression in control regions, i.e. anterior ventral part of the H, IH and MD (Fig. 2A; relative mean pixel density: day: −20 ± 24, night: 72 ± 12; *t*-test: d.f. = 8, *t* = −6.85, *P* < 0.001; Mann–Whitney *U*-test: *n*_1_ = 6, *n*_2_ = 4, *U* = 24, *P* = 0.01). The control regions showed no significant difference between the day- and night-group (Mann–Whitney *U*-test: *n*_1_ = 6, *n*_2_ = 4, day median 78, night median 46, *U* = 20, *P* = 0.11). To exclude that differences in pixel density and therefore differences in ZENK expression could be due to variations in the staining process and/or photo-imaging, we also measured pixel density in the globus pallidus, a brain area known not to express ZENK, and found no differences between groups (day: 29 ± 11; night 25 ± 10, *t*-test, d.f. = 8, *t* = 0.576, *P* = 0.58).

Different levels of ZENK and GluR1 expression in the DNH and DNH-shell have been described previously in European robins and garden warblers (Mouritsen *et al.*, 2005; Heyers *et al.*, 2007). These obvious neuroanatomical characteristics of Cluster N were also prominent in the day- and night-migrating meadow pipit: the DNH showed high expression of GluR1 (Fig. 1E) and lower expression of ZENK compared with the surrounding DNH-shell when Cluster N was ZENK positive. Comparing the neuronal activation patterns of day- and night-migratory species, the same brain regions are activated as a columnar unit (i.e. H, IH, MD, DNH and DNH-shell) and we therefore conclude that meadow pipits do also have a Cluster N.

During the day, and in contrast to the night-time activation in Cluster N, consistent increases in expression occurred in a set of regions surrounding the entopallium (Fig. 2A). The entopallium receives thalamic visual input from the tectofugal pathway, and like other primary sensory telencephalic neurons it does not show prominent ZENK expression (Mello & Clayton, 1995). However, the visual centres around the entopallium (entopallial belt area) do show ZENK induction in response to light stimulation (Feenders *et al.*, 2008; Hara *et al.*, 2009; Horita *et al.*, 2010). The daytime activated regions around the entopallium included a portion of the nidopallium and a portion of the ventral mesopallium, forming a ventral to dorsal column of brain activation that is part of the tectofugal visual pathway (Colombo *et al.*, 2001; Krützfeldt & Wild, 2004; Feenders *et al.*, 2008).

## Discussion

### Cluster N may be a general feature of migratory passerines migrating at least partly during the night

The neuronal activation patterns in the brain of day- and night-migrating meadow pipits showed that meadow pipits have a forebrain region that, based on its location and biochemical characteristics, corresponds to Cluster N in night-migratory birds. In day- and night-migrating meadow pipits, this brain area shows high levels of neuronal activation during night-time, but not during the day.

Given that (i) Cluster N is connected to the retina via the thalamofugal visual pathway and is thus part of the visual Wulst in birds (Heyers *et al.*, 2007), (ii) the thalamofugal pathway terminates in the visual Wulst (for a review see Güntürkün *et al.*, 1993; Güntürkün, 2000) and (iii) an intact Cluster N is required for magnetic compass orientation in a night-migratory songbird (Zapka *et al.*, 2009), our findings indicate that meadow pipits possess a Cluster N, and that Cluster N may be present in all migratory songbirds. Based on our findings we also speculate that night- and/or day-migratory birds might share the same vision-mediated mechanism underlying magnetoperception, at least during the night.

### Meadow pipits may use compass cues other than the magnetic field during day-migration

Our finding of a functional Cluster N in the meadow pipit implies that this day- and night-migrating species is putatively able to detect the Earth’s magnetic field, and might use this information as a compass reference cue. This suggestion is supported by behavioural experiments where several diurnal or partly diurnal species [e.g. yellow-faced honeyeaters (*Lichenostomus chrysops*; Munro & Wiltschko, 1993), sanderlings (*Caladris alba*; Gudmundsson & Sandberg, 2000), barn swallows (*Hirundo rustica*; Giunchi & Baldaccini, 2004), domestic chicken (*Gallus gallus*; Freire *et al.*, 2005), tree pipits (*Anthus trivialis;* Åkesson *et al.*, 2006)] have been successfully tested for their ability to use the magnetic field as a reference system during the day.

Surprisingly, Cluster N of meadow pipits only showed high neuronal activation during the night and dim light conditions but not during the day. What does this mean? One possible explanation might be that, during the day, meadow pipits preferably use another magnetosensory mechanism and/or (an-) other reference system(s) like the sun and/or polarization pattern of the sky. The results of Helbig *et al.* (1987), who tested meadow pipits in Emlen funnels for their orientation abilities under different experimental conditions during the day, provide some support for this idea. Under clear skies in the natural magnetic field, the birds were able to orientate in their appropriate migratory direction, whereas the same birds were disorientated under overcast conditions. This indicates either that the meadow pipits did not use their magnetic compass to orientate in their migratory direction during the day or that they were not motivated to migrate under overcast conditions (Helbig *et al.*, 1987). Another explanation of our data could be that Cluster N was also active during the day, but ZENK was not induced there. If true, then it would suggest different molecular or activity responses in Cluster N during the day, and thus different functional processing in Cluster N during night and day migration. That is, either of these mechanisms leads to the same conclusion, that Cluster N is differentially regulated in night- vs. daytime migration.

The polarization pattern is highly regular depending on the position of the sun and it is regularly distributed throughout the globe (Cronin & Shashar, 2001). Even if the sky is not thickly overcast, light passes through the clouds and the polarization pattern is conserved and provides an accurate reference source (Pomozi *et al.*, 2001). Therefore, the polarized light pattern might serve as the primary reference system for compass orientation in meadow pipits during the day (e.g. Munro & Wiltschko, 1995; Muheim *et al.*, 2006).

### Implications for the vision-mediated magnetic compass

Helbig *et al.* (1987) showed that, in the wild, more than 75% of free-flying meadow pipits, which were counted within 6 h after sunrise, migrated during the first 3 h after sunrise. The spectral pattern of the ambient light changes during the day (Cronin & Shashar, 2001) and it might be possible that a certain light spectrum and/or light intensity is required for magnetic compass orientation based on the light-dependent mechanism in the eye of a given species of bird (e.g. Wiltschko *et al.*, 1993, 2007; Wiltschko & Wiltschko, 2001; Muheim *et al.*, 2002).

Furthermore, there seems to be a strong correlation between the brightness of the ambient light and orientation abilities: several species of night-migratory songbirds tested under higher light intensities were disorientated, and a correct migratory direction was only chosen under lights with irradiances below that of sunset (for a review see Johnsen *et al.*, 2007). Consequently, the light regime could have been too bright, i.e. outside the ‘functional window’ (Johnsen *et al.*, 2007; Wiltschko *et al.*, 2007) of the birds’ magnetic compass when we tested our birds during daytime under full room light (250 mW/m^2^). It should be noted that few if any well-orientated data from night-migratory passerines tested in orientation cages under bright sunlight conditions exist. Even though a bimodal light intensity sensitivity curve for the involved light-dependent magnetic sensors cannot be completely excluded, it seems highly unlikely that the cryptochromes or any other potential light-dependent magnetosensor would be sensitive at very dim light and at very high light intensities, but not at intermediate light intensities.

The possibility that an upper light-intensity limit means that the light-dependent magnetic compass of at least partly night-migrating songbirds may only work during the night could easily be understood from a physiological perspective. If a cryptochrome is the primary magnetoreceptor (Ritz *et al.*, 2000; Möller *et al.*, 2004; Mouritsen *et al.*, 2004a; Liedvogel *et al.*, 2007b; Maeda *et al.*, 2008), then cryptochromes located in the ganglion cells and/or in the photoreceptor cells of the eye are good candidates (Mouritsen *et al.*, 2004a). Both of these types of retinal neurons are also involved in normal visual processing. Consequently, if the light-dependent magnetic compass detection mechanism uses, at least in part, the same cell types and transduction pathways that are used for normal daytime vision, it is possible that normal day-vision processes mask or override light-dependent magnetic compass information during the day, especially in night-migratory birds that could easily have a magnetic compass detection mechanism optimized for night-time use. If the relevant primary sensory molecules underlying light-dependent magnetic compass sensing turns out to be located in one or more types of retinal cones, the masking hypothesis becomes particularly likely: the cones are most strongly involved in processing normal visual information, including colour vision during the day, whereas the cones are generally thought not to be involved in low-light vision. Therefore, the cone pathways would be ‘free’ for use in magnetic compass sensing at night.

Another hypothesis (Muheim *et al.*, 2002) speculates that two magnetically sensitive, interacting spectral mechanisms could be involved: a dominant light-dependent receptor operating in the blue–green range of the spectrum and a secondary mechanism requiring either long wavelengths or no light at all. It is possible that these two mechanisms act antagonistically under light intensities that are too bright and that this can explain why a light-dependent magnetic compass putatively adapted to work primarily during the night might not be functional during the day. Nevertheless, it is possible that other species that navigate exclusively during the day, such as the homing pigeon, have evolved a light-dependent magnetic compass sensitivity, which is adapted to functioning well during the day.

## Conclusions

A forebrain region, which corresponds to Cluster N in purely night-migratory songbirds, shows strong neuronal activation during the night in meadow pipits. This raises the possibility that, in songbirds migrating at least partly during the night, Cluster N might only process magnetic compass information during the night. Our findings thus support the view of Johnsen *et al.* (2007) that the light-dependent magnetic compass mechanism may only function below a certain light intensity threshold in night-migratory songbirds. These findings could potentially be explained by the dominance of daytime vision over magnetic compass signals, particularly if the light-dependent magnetosensory molecules were located in cones.

## Abbreviations

DNH: dorsal nucleus of the hyperpallium
GluR1: glutamate receptor type 1
H: hyperpallium
IEG: immediate early gene
IH: interstitial region of the hyperpallium
MD: dorsal mesopallium
PBS: phosphate-buffered saline
PFA: paraformaldehyde
